# The emergence, maintenance, and demise of diversity in a spatially variable antibiotic regime

**DOI:** 10.1002/evl3.43

**Published:** 2018-03-17

**Authors:** Alanna M. Leale, Rees Kassen

**Affiliations:** ^1^ Department of Biology University of Ottawa Ottawa Ontario K1N 6N5 Canada

**Keywords:** Evolutionary medicine, experimental evolution, microbes, trade‐offs

## Abstract

Antimicrobial resistance (AMR) is a growing global threat that, in the absence of new antibiotics, requires effective management of existing drugs. Here, we use experimental evolution of the opportunistic human pathogen *Pseudomonas aeruginosa* to explore how changing patterns of drug delivery modulates the spread of resistance in a population. Resistance evolves readily under both temporal and spatial variation in drug delivery and fixes rapidly under temporal, but not spatial, variation. Resistant and sensitive genotypes coexist in spatially varying conditions due to a resistance‐growth rate trade‐off which, when coupled to dispersal, generates negative frequency‐dependent selection and a quasi‐protected polymorphism. Coexistence is ultimately lost, however, because resistant types with improved growth rates in the absence of drug spread through the population. These results suggest that spatially variable drug prescriptions can delay but not prevent the spread of resistance and provide a striking example of how the emergence and eventual demise of biodiversity is underpinned by evolving fitness trade‐offs.

**Impact Summary**

The rise of antimicrobial resistance among human pathogens is one of the most pressing global health challenges. In the absence of novel drugs, we urgently need strategies to manage our existing arsenal of drugs. We show experimentally that drug sanctuaries in space, but not time, can slow the spread of resistance by promoting the coexistence of resistant and sensitive strains through negative frequency‐dependent selection (where genotypes have higher fitness when rare than when common). These results are consistent with models of selection in variable environments that predict spatially variable environments are more effective at supporting diversity than temporally variable ones. Coexistence is eventually undermined by selection, however, as resistant genotypes with reduced costs evolve to displace sensitive genotypes. Taken together, our results suggest that drug sanctuaries in space can delay but not prevent the spread of resistance. They also provide insight into the mechanisms governing diversity on evolutionary time scales. More generally, our work underscores the value of using evolutionary principles to inform public health intervention strategies and the utility of in vitro microbial evolution for evaluating these ideas through proof‐of‐principle experiments.

The effectiveness of antibiotic therapy to control infection is being steadily undermined by the combination of divestment in drug discovery, widespread occurrence of genetic resistance among microbes isolated from natural environments (Nesme et al. 2014), and continued evolution of resistant strains among all major pathogens (Hidron et al. 2008). Managing our existing arsenal of drugs to ensure they remain effective for as many people for as long as possible is therefore an urgent public health priority.

Beyond a blanket ban on prescriptions, there is little consensus on how best to delay or prevent the emergence and spread of resistance. Using multiple drugs with distinct cellular and genetic targets is often suggested as the most effective treatment but sequential use can often select for multidrug resistant strains (Alekshun and Levy 2007), while simultaneous delivery (as a drug cocktail) can be difficult for patients to tolerate (Tamma et al. 2012). An alternative approach is to exploit the use of drug‐free sanctuaries or refuges in time or space to make it more difficult for selection to fix resistant strains. Sensitive strains are expected to outcompete resistant strains that pay a fitness cost of resistance (Melnyk et al. 2015) in drug sanctuaries, implying that resistance could be kept at manageably low levels if dispersal from sanctuaries introduces sensitive strains at a sufficiently high rate (Felsenstein 1976; Andow and Alstad 1998; Austin et al. 1999). Sanctuaries have been used to good effect in managing resistance in agricultural systems (Hutchison et al. 2010; Carrière et al. 2012) but their role in governing the spread of resistance in health settings remains unclear (Debarre et al. 2009; Park et al. 2015).

Theory suggests that the way sanctuaries are experienced in time and space can profoundly impact resistance evolution (Felsenstein 1976; Bonhoeffer et al. 1997; Bergstrom et al. 2004; Debarre et al. 2009; Kassen 2014). Periodic delivery of a drug in time generates regular cycles of strong antibiotic selection followed by periods of relaxed selection when the drug is either metabolized, excreted, or not in use (Mackenzie and Gould 1993; Mouton and Vinks 1996). Intermittent dosing or ward‐wide use generates fluctuating selection that leads to the evolution of broadly adapted resistant types with high fitness in both the presence and absence of drug (Melnyk et al. 2017). Drug delivery may also be spatially variable across wards in a hospital or among compartments (tissues or organs) within a host, generating divergent selection that can lead to the emergence of a single resistant generalist or, if selection is sufficiently strong relative to dispersal (which can happen, e.g., through the movement of patients and staff between hospital wards), the coexistence of more narrowly adapted sensitive and resistant genotypes that trade‐off drug resistance with growth rate in the absence of drug (Felsenstein 1976; Debarre et al. 2009; Kassen 2014).

Empirical evidence bearing on these predictions remains sparse. Drug sanctuaries in time have been shown to select for cost‐free, drug resistant genotypes (Melnyk et al. 2017) while spatial gradients in drug delivery can lead to fairly rapid evolution of resistance through selection for partially resistant intermediate mutants (Zhang et al. 2011; Baym et al. 2016). There is a substantial and growing literature devoted to evaluating different multiple‐drug delivery options using in vitro evolution (reviewed in Maclean et al. 2010 and Palmer and Kishony 2013; Kim et al. 2014; Fuentes‐Hernandez et al. 2015) on the rate of resistance evolution. Little attention has been paid, however, to how drug sanctuaries impact the spread of resistance through a population, the evolution of trade‐offs between resistance and sensitivity, and the potential for coexistence between resistant and sensitive types.

To evaluate the impact of drug sanctuaries on the emergence and spread of AMR, we tracked the evolution of resistance and fitness in 12 independently evolved, isogenic lines of *Pseudomonas aeruginosa* strain PA14 propagated in environments that varied through time or space with subinhibitory concentrations of the commonly used fluoroquinolone antibiotic, ciprofloxacin. *P. aeruginosa* ranks among the top three drug resistant pathogens globally (World Health Organization 2017) and causes both acute infections of wounds and chronic infections of the respiratory tract, where it is a major source of morbidity and mortality in adults with cystic fibrosis (CF) (UK CF Trust Antibiotic Working Group 2009; Folkesson et al. 2012). We use drug concentrations that resemble that found in the sputum of CF patients undergoing fluoroquinolone treatment during exacerbations, which are periods of acute decrease in lung function accompanied by an increase in microbial (often *P. aeruginosa*) population density (Pedersen et al. 1987). Controls were a permissive (PERM) environment consisting of drug‐free Luria‐Bertrani (LB) medium and a constant selective environment (CONS) comprised of LB supplemented daily with subinhibitory concentrations (0.3 μg/mL) of ciprofloxacin sufficient to reduce population densities to 20% of that in the absence of drug. Temporally varying environments (TEMP) were constructed by transferring aliquots from each evolving population into either permissive (no drug) or selective (0.3 μg/mL) conditions on alternating days. Spatially variable environments (SPAT) consisted of two subpopulations, or patches, one permissive and one selective, with equal volume aliquots from each mixed and redistributed into fresh drug‐free and drug‐supplemented media daily (see Methods and Fig. S1). Note that the SPAT treatment contains, for any given lineage, a degree of temporal heterogeneity since the probability of a lineage experiencing an environment different from the one in which it currently resides in the next growth cycle is 0.5. The SPAT treatment resembles a well‐known model in population genetics in which genetic polymorphism can be maintained through negative frequency‐dependent selection, provided there is a strong fitness trade‐off among genotypes across patches and population regulation occurs at the level of the local patch rather than the total population (Levene 1953; Dempster 1955; Kisdi 2001; Debarre and Gandon 2011). However, the transfer protocol used in our SPAT treatment involves mixing samples by volume, and thus more closely resembles the “hard selection” version of the model that is not expected to lead to stable coexistence (Dempster 1955; Christiansen 1975). In contrast to previous work (Reboud and Bell 1997; Jasmin and Kassen 2007; Huang et al. 2014), fitness trade‐offs evolve naturally over the course of the experiment.

## Results and Discussion

**Figure 1:**
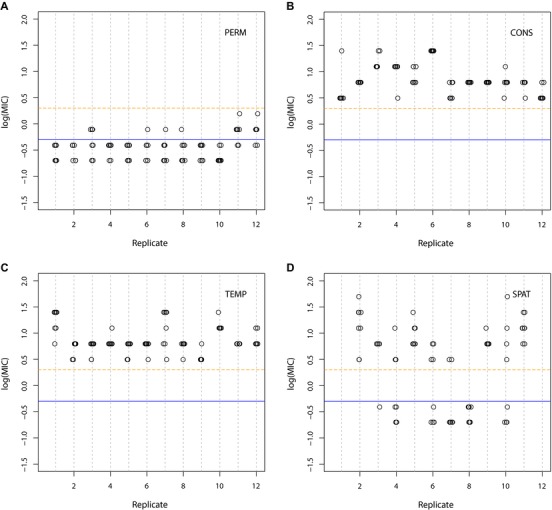
Resistance, measured as the log minimum inhibitory concentration (log_10_(MIC), with MIC measured in μg/mL) of each of eight isolates from each evolved population after 20 days of serial transfer (∼133 generations). (A) PERM, (B) CONS, (C) TEMP, and (D) SPAT. Blue line indicates resistance level of ancestral PA14 isolate (MIC = 0.5 μg/mL, log_10_(MIC) = –0.3). An isolate is deemed resistant if its MIC > 2 μg/mL (log_10_(MIC) = 0.3), as indicated by the orange dashed line. Each data point represents a single isolated colony.

After 20 days (∼133 generations) of selection, resistance (defined as a minimum inhibitory concentration–‐or MIC, the lowest drug concentration that completely inhibits growth–‐of >2 μg/mL) failed to evolve in the absence of drug selection (Fig. 1A) but did evolve under all other conditions where ciprofloxacin was present (Fig. 1B–D). The level of resistance was assayed by determining the MIC for eight randomly selected colonies from each evolved population. Resistance fixed in all populations experiencing drug sanctuaries in time (Fig. 1C) but not space, where 6 of 10 populations contained a mixture of resistant and sensitive colonies (Fig. 1D; note that two populations in this treatment were lost due to contamination). Notably, the least resistant genotypes in the spatially structured environment (Fig. 1D) were as sensitive to ciprofloxacin as the ancestor and evolved populations from the permissive environment (Fig. 1A), while the most resistant genotypes had MICs similar to evolved genotypes from both the constant (Fig. 1B) and temporally varying (Fig. 1C) environments. Thus, drug sanctuaries do little to prevent the initial evolution of resistance but, when spatially structured, can slow the rate at which resistance sweeps through the population. More generally, divergent selection caused by spatial heterogeneity in antibiotic concentrations can promote diversification and coexistence between resistant and sensitive genotypes, while fluctuating selection on a similar time‐scale does not.

**Figure 2:**
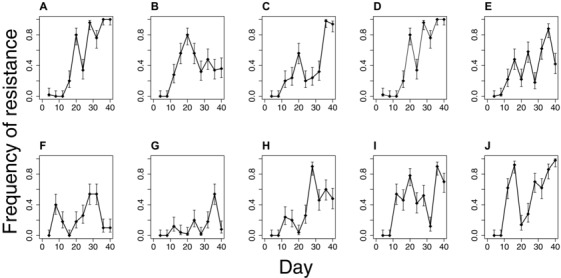
Dynamics of resistance within all SPAT populations. Each panel (A–J) is an independently evolved population. Sensitive colonies were detected at all‐time points in all populations except populations C, D, and J where all 50 colonies were resistant by day 36 and/or 40. Each data point represents a single estimate of frequency, with binomial confidence intervals (alpha = 0.05), as determined from assaying 50 randomly selected colonies.

**Figure 3:**
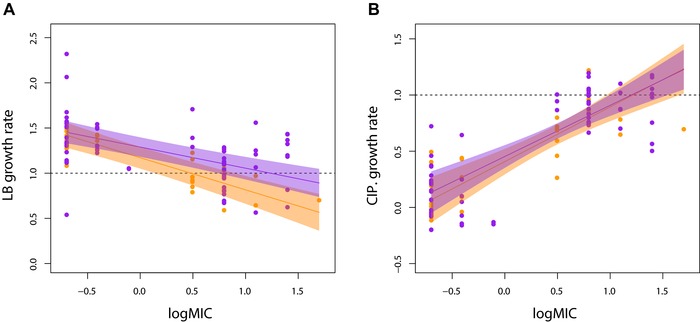
The trade‐off between resistance and growth rate in LB (A) and LB supplemented with 0.3 μg/mL ciprofloxacin (B). Data is standardized to the growth rate of the ancestral PA14 in LB (horizontal dashed lines in each panel). Shaded areas represent 95% confidence intervals for day 20 (orange) and day 40 (purple). Only populations with both resistant and sensitive isolated colonies were included for analyses.

**Figure 4:**
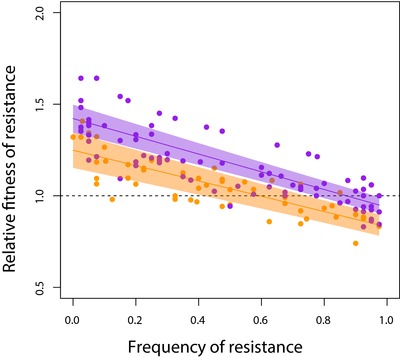
Negative frequency‐dependent selection between resistant and sensitive strains within populations. Fitness of a resistant colony relative to a paired sensitive colony (ω) is plotted as a function of its starting frequency. Shaded areas represent 95% confidence intervals for day 20 (orange) and day 40 (purple). The frequency of resistance at equilibrium is given by the intersection of the regression line with relative fitness (ω) = 1. Only populations with both resistant and sensitive isolated colonies were included for analyses with four random pairs of resistant and sensitive colonies assayed from each population. Each data point represents a single estimate of fitness (day 20 total = 57 data points, day 30 total = 156 data points).

Does the diversity in resistance profiles in the spatially variable environment reflect stable coexistence, or might it simply be a transient effect reflecting a reduced overall strength of selection for resistant types (Whitlock 2003)? Four lines of evidence point to diversity being stably, or at least quasi‐stably, maintained in the SPAT populations. First, coexistence between resistant and sensitive strains persists through an additional 20 days of selection in 8 of 10 populations from the SPAT treatment, albeit with marked fluctuations through time (Fig. 2). Second, we did not observe any evidence of sensitive genotypes persisting under temporally variable conditions, as might be expected if the primary effect of the permissive patch is to slow the rate of fixation of resistance mutations (Fig. S2). Third, we observed the expected trade‐off between resistance and growth rate in the absence of drug for the eight previously isolated colonies at day 20 and an additional eight colonies isolated at day 40 (Fig. 3A; mixed linear analysis of covariance between growth in LB and log_10_(MIC)*day, with population as a random effect: overall effect of log_10_(MIC) tested by model comparison = –0.2756, *χ* ^2^ = 22.1312, *P* < 0.0001; results from the full model presented in Table S1). Fourth, and most convincingly, invasion from rare experiments (see Methods) between four randomly selected pairs of resistant and sensitive isolates from SPAT populations at day 20 and day 40 show that rare genotypes always have higher fitness than common ones, providing direct evidence for negative frequency‐dependent selection (Fig. 4; mixed linear analysis of covariance between relative fitness (ω) and day × resistance frequency, with random effects of individual isolate pair nested in population: slope for main effect of resistance frequency tested by model comparison = –0.4584, *χ* ^2^ = 45.8185, *P* < 0.0001; full model results in Table S2). Notably, this result is robust to how we estimate the initial frequency of resistance (see Fig. S7 and Table S4), suggesting that sampling error cannot be solely responsible for the observed negative slope.

These results suggest that divergent selection leads to the coexistence of resistant and sensitive strains through negative frequency‐dependent selection, consistent with predictions from models for the maintenance of polymorphism in spatially variable environments where population regulation occurs at the level of the local patch (Levene 1953; Kisdi 2001). This result is surprising because our transfer protocol, which regulates population size at the level of the total population, is not expected to lead to coexistence via negative frequency‐dependent selection (Dempster 1955). It may be that the transfer protocol actually permits some degree of patch‐level population regulation, a scenario that has been shown to broaden the conditions for coexistence (DeMeeûs and Goudet 2000). When coupled with changes to the relative productivity of drug‐containing and drug‐free patches that make them more similar by days 20 and 40 compared to the start of the experiment (Fig. S4), the conditions for stable coexistence through negative frequency‐dependent selection become easier to satisfy (Kassen et al. 2000).

**Figure 5:**
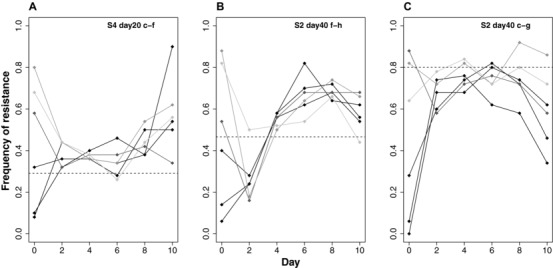
Dynamics of resistant‐sensitive pairs starting from different starting frequencies. The six shades of gray within each panel depict how the frequency of a resistant strain, relative to a sensitive strain from the same population, changes when initiated from a different starting ratio. Each panel (A–C) refers to a different resistant‐sensitive pair of strains isolated from the original experiment. Example: “S4 day 20 c‐f” refers to day 20, SPAT population 4, isolates c and f. Each data point represents a single estimate of frequency, as determined from assaying 50 randomly selected colonies.

Despite the presence of strong negative frequency‐dependent selection, the polymorphism does not appear to be stable in our populations on evolutionary time scales. Coexistence persists at day 40 in just three populations (Fig. 2A, B, H) whereas four populations are fixed or nearly fixed for resistance (Fig. 2C, D, I, J) and nearly lost in two (Fig. 2F, G). The fixation or loss of resistant strains could be due to stochastic variation in the frequency of resistant types, perhaps associated with the daily serial transfer protocol we imposed in our experiment, or from high mutation supply rates leading to complex dynamics generated from competition among beneficial mutations that arise independently on different genetic backgrounds within the same population (clonal interference). To distinguish between these alternatives, we tracked the frequency of the resistant strain in three pairs of resistant and sensitive isolates over time under spatially variable conditions (see Methods). In the absence of clonal interference, we expect the frequency of resistance to tend toward an internal equilibrium without fluctuations, regardless of starting frequency. As expected, the frequency of resistant genotypes converges toward an equilibrium point between 0 and 1 within the first two days and remains relatively stable until day 8, after which frequencies diverge again, presumably as mutations are reintroduced into the population (Fig. 5). These results suggest that the fluctuations in the frequency of resistant and sensitive strains we observed in our original experiment are likely due to high mutation supply rates generating clonal interference (see also Maddamsetti et al. 2015), rather than stochastic effects associated with our transfer protocol.

High levels of clonal interference imply that there is a steady source of genetic variation on which selection can act, over and above the diversity supported by negative frequency‐dependent selection. If so, are these populations continuing to evolve or does negative frequency‐dependent selection act to prevent further evolution? To answer this question, we focused attention on the how the trade‐off between MIC and growth rate under drug‐free conditions changed over the selection experiment. We find that the slope of this trade‐off evolves to become shallower at day 40 than at day 20 (Table S1; significance of log_10_(MIC)*day interaction tested by model comparison: *χ* ^2^ = 4.171_,_ *P* = 0.0411). Inspection of Figure 3A suggests that this effect is due to increases in the growth rate of resistant isolates in LB, rather than loss of resistance (Fig. 3A). Further support for this interpretation comes from the lack of change in growth rate of resistant isolates in the presence of ciprofloxacin between days 20 and 40 (Fig. 3B, Table S3; log_10_(MIC)*day interaction from model comparison: *χ* ^2^ = 0.2112, *P = *0.6459). This result is consistent with the selection of second‐site compensatory mutations that improve growth rate under drug‐free conditions without compromising resistance (Gagneux et al. 2006; Comas et al. 2012; Wong et al. 2012; Melnyk et al. 2017) or the selective replacement of costly resistance mutations by independently‐arising resistance mutations with lower costs. Irrespective of the underlying genetic causes of this weakened trade‐off, the internal equilibrium frequency of resistant strains (i.e., resistance frequency when ω = 1) increases on average from ∼59% at day 20 to ∼87% by day 40 (Fig. 4), implying that resistant genotypes are slowly spreading through most populations. As before, similar results obtain if we use the volumetric ratios as estimates of initial frequency of resistance, suggesting that sampling error contributes little to this result. A putatively stable polymorphism on ecological time scales can thus be readily undermined by selection which, in this case, leads to the evolution of broadly adapted genotypes that are both resistant and have high fitness under drug‐free conditions and the eventual loss of sensitive genotypes.

## Conclusion

Our results paint a rather pessimistic picture for the prospect of using drug sanctuaries to manage antimicrobial resistance. Given the strong selection generated by widespread antibiotic use, drug sanctuaries of the sort we have studied here cannot prevent the evolution of drug resistance. Nevertheless, the manner in which drug sanctuaries are experienced by a pathogen can impact the rate at which resistance spreads in a population: while temporal variation in drug sanctuaries does little to prevent the rise of resistance, spatially distributed sanctuaries can slow the rate at which resistance fixes in a population. In line with the predictions of evolutionary theory, the strong divergent selection imposed by spatial variation in drug delivery leads to the emergence of a genetic polymorphism between resistant and sensitive strains supported by negative frequency‐dependent selection. In contrast with classic models of selection in spatially variable environments, however, this polymorphism is quasi‐stable in the long‐term, being readily undermined by the evolution of generalist resistant strains with high fitness in the absence of drugs. In other words, even spatial variation in drug selection cannot prevent the eventual loss of sensitive strains due to the evolution of drug resistant strains with reduced costs of resistance.

How general are our results? An answer will have to await further tests. We have focused on just one pathogen evolving in response to one drug under drug dosing regimens that are meant, by design, to capture the essential features of temporal and spatial variation. The advantage of this approach is that it allows us to ask focused questions about the impact of these idealized forms of environmental variation on the evolution and spread of resistance and also directly tie the results to long‐standing theory on selection in variable environments. The disadvantage, of course, is that our experiments are a far cry from the more complex environments and dynamics of pathogen populations in the real world. Obvious next steps will be to explore the efficacy of sanctuaries in managing resistance across a wider set of pathogen‐drug combinations and under a broader set of environmental conditions that more closely resemble how pathogen populations experience drugs in the host, clinic, or community.

If our results do prove to be general, then the implication here should be clear: in the absence of a continual pipeline of new drugs for treating infectious disease, the best we can do is slow the rate at which existing drugs lose efficacy. The use of drug sanctuaries in space may help, for a time, but natural selection will almost certainly find a way to undermine our best strategies for preventing resistance. The problem is further exacerbated by the fact that drug sanctuaries, which can support large populations due to the absence of drug, can serve as reservoirs of resistant variants generated by mutation (Kepler and Perelson 1998; Perron et al. 2007; Zhang et al. 2011; Hermsen et al. 2012). If on the other hand, as has been suggested by Maclean and Vogwill (2015), compensatory mutations that alleviate the cost of resistance occur only rarely in clinical settings, then drug sanctuaries in space could still be effective at delaying the fixation of resistant types, though perhaps not preventing their initial emergence. The important point here is that drug resistance management strategies should take evolutionary principles into account to, in an ideal world, minimize the opportunity for resistance both to evolve and to spread. If drug sanctuaries cannot prevent the emergence of resistance, either because resistant strains are widespread in nature or they evolve readily via mutation, they may still be useful tools for managing its prevalence.

## Methods

### EXPERIMENTAL EVOLUTION

A single colony of *P. aeruginosa* strain PA14 was grown overnight in Luria‐Bertrani broth (LB: bacto‐tryptone 10 g/L, NaCl 10 g/L, yeast extract 5 g/L) and used to found 48 populations by adding 20 μL into 1.5 mL of fresh media (described below). An aliquot of the progenitor was frozen in glycerol at –80°C. Every 24 hours, a 20 μL aliquot of overnight culture was added to 1.5 mL of fresh medium. Populations were propagated in 24‐well microtiter plates and agitated using an orbital shaker (150 rpm) at 37°C. Samples were frozen at –80°C in glycerol every four and ten transfers, or approximately every 26 and 66 generations, respectively.

The experiment consisted of 12 replicate populations for each of four treatments growing in LB broth supplemented or not with 0.3 μg/mL of the fluoroquinolone antibiotic, ciprofloxacin, for 40 days, or approximately 265 generations. This concentration of ciprofloxacin inhibits growth of the sensitive PA14 ancestor to approximately 20% of full growth in LB over a 24‐hour period. Treatments were: (A) a permissive environment involving daily propagation in drug‐free LB (PERM); (B) a constant environment with ciprofloxacin added daily (CONS); C) a temporally varying environment consisting of daily alternation in drug‐free and drug‐supplemented media (TEMP); and D) a spatial treatment comprised of two subpopulations, one containing drug and the other drug‐free, connected through dispersal (SPAT). Dispersal was imposed by mixing 0.75 mL from each pair of wells prior to transfer and inoculating a fresh pair of wells with 20 μL of the combined mixture (see Fig. S1). Samples of the mixed population were frozen. Two populations from the SPAT treatment were excluded from final analyses due to contamination.

### MINIMUM INHIBITORY CONCENTRATION (MIC) AND GROWTH RATE

We randomly selected eight colonies (named “a” to “h”) from each population at day 20, plus an additional eight colonies from the TEMP and SPAT treatments at day 40, by plating a sample of each population on agar and choosing those closest to an arbitrary point in the middle of the plate. For each isolate, we assayed resistance as the MIC to ciprofloxacin by first reviving frozen cultures overnight in LB media and then inoculating 100 μL of dense culture into 96‐well plates containing LB supplemented with 0.0, 0.10, 0.20, 0.39, 0.78, 1.56, 3.12, 6.25, 12.5, 25, 50, or 100 μg/mL ciprofloxacin, respectively, and incubating on an orbital shaker at 37°C. Growth was scored by reading optical density (OD) at 600 nm after 48 hours. Log‐transformed MICs were used for all analyses. Resistant strains were defined as those having an MIC exceeding 2 μg/mL, equivalent to 4x the ancestral MIC (0.5 μg/mL). Results are shown in Figures 1 and S2.

The growth of evolved SPAT isolates relative to that of PA14 was measured in LB with and without ciprofloxacin over 24 hours. In LB, 5 μL of overnight culture was added to 195 μL of LB and OD600 measured every 90 minutes. The same procedure was performed for ciprofloxacin growth assays, except we used 20 μL of overnight culture with 180 μL of LB‐ciprofloxacin media (0.3 μg/mL). Gen5 software (BioTek Instruments Inc., Winooski, VT) was used to estimate maximum growth rate during exponential phase for three replicates per isolate. Growth rates are expressed relative to the mean growth rate of PA14 in LB when grown on the same 96‐well assay plate. Results are depicted in Figure 3.

### NEGATIVE FREQUENCY‐DEPENDENT SELECTION

Using reciprocal competitive invasion experiments, we estimated the strength of negative frequency‐dependent selection between resistant and sensitive isolates from all SPAT treatment populations that showed evidence of coexistence from the MIC assays. We first chose four random pairs of resistant and sensitive colonies from each population, then competed each pair by mixing pure culture samples from overnight cultures at ratios of 1:9, 1:1, and 9:1 by volume and allowing them to compete for two transfers (48 hours) under the same SPAT treatment transfer protocol as in the original selection experiment. We tracked the change in relative abundance of sensitive and resistant colonies by streaking 40 randomly selected colonies on 2 μg/mL ciprofloxacin agar for both the initial (0 h) and final (48 h) populations. Relative fitness, ω, was estimated using the equation:

ω=f final f initial ^1 doublings

where *f*_initial_ and *f*_final_ are the odds ratio of resistance before and after competition, respectively (i.e. the ratio of the frequency of resistance types to the frequency of sensitive types in the population). Doublings refers to the number of generations occurring between the initial and final measurements (∼13 generations). We note that this formula assumes fitness is constant and therefore represents an approximation to the true fitness across a given frequency interval. Results are shown in Figure 4.

We omitted four R–S pairs from four populations (one each from day 20—populations S3 and S10, and day 40—populations S8 and S9) due to experimental error. If the frequency of resistance was 0 or 1 (i.e., 0/40 or 40/40), the value was adjusted to be 1/40 or 39/40 to allow the data point to still be included in analyses. This correction is conservative as it underestimates the true fitness value.

### DYNAMICS OF RESISTANCE

We assayed the frequency of resistance every four days for each replicate of the SPAT treatment by isolating 50 colonies at random on LB plates, streaking each colony on 2 μg/mL ciprofloxacin agar, and visually inspecting each plate for growth after 24 hours at 37°C. Results shown in Figure 2.

### DYNAMICS OF INVASION FROM RARE

We chose one sensitive and one resistant isolate (a “pair”) from population S4 at day 20 (isolates c and f) and two pairs from population S2 at day 40 (isolates f and h, and c and g) to investigate the dynamics of resistance in the absence of clonal interference. These pairs were chosen because preliminary experiments indicated each had a different predicted equilibrium frequency (i.e., the frequency of resistance where ω = 1 in reciprocal competitive invasion assays). Pairs of isolates were grown overnight as pure cultures and initial ratios of resistant and sensitive strains were constructed at 1:9, 2:8, 4:6, 6:4, 8:2, and 9:1 by volume, then mixtures were propagated identically to that in the original SPAT treatment for 10 days (∼65 generations). Founding populations (day 0) and evolved populations were archived daily. The frequency of resistant individuals over time was estimated by testing the presence or absence of growth of 50 randomly selected colonies streaked on 2 μg/mL ciprofloxacin agar, as described above, at days 0, 2, 4, 6, 8, and 10. Results are shown in Figure 5.

### STATISTICAL ANALYSIS

All statistical analyses were conducted using R statistical software (R Core Team 2016, Version 3.3.1; https://www.r-project.org). We used mixed linear analyses of covariance to model the trade‐off between maximum growth rate in drug‐free media as a function of the fixed effect of log_10_(MIC) and day, with population treated as a random effect: $maxV=log_{10}\;{\left(\mathrm{MIC}\right)}^{*}day\;+\left(log_{10}\left(MIC\right)|\mathrm{population}\right)$. An interaction term between log_10_MIC and day was included to determine whether the fitness trade‐off differed between day 20 and 40. A model comparison approach was used to calculate the significance of the interaction effect. A similar approach was used to estimate the frequency‐dependent fitness functions, with relative fitness (ω) modeled as a function of the fixed effects of day and initial frequency of resistance (*F*_initial_) with random effects of isolate pair nested in population: ω=F initial ×day+(F initial |( population : pair )). Evidence that coexistence is supported by negative frequency dependence exists if there is both a) statistically significant negative slope between fitness and frequency of resistance, and b) the regression line crosses the relative fitness axis (i.e., ω = 1) between at a frequency between 0 and 1. Note that the *x*‐intercept additionally provides an estimate of the location of the internal equilibrium frequency of resistance.

Associate Editor: Z. Gompert

## Supporting information


**Table S1**. Mixed linear analysis of covariance for maximum growth rate in LB.
**Table S2**. Mixed linear analysis of covariance for relative fitness (ω) of resistant types, with random effect of isolate pair nested in population.
**Table S3**. Mixed linear analysis of covariance for maximum growth rate in [0.3 μg/mL] ciprofloxacin.
**Table S4**. Mixed linear analysis of covariance for relative fitness (ω) of resistant types, with random effect of isolate pair nested in population.

**Fig. S1**. SPAT selection regime.

**Fig. S2**. Coexistence of susceptible and resistant types maintained in SPAT treatment.

Supplementary Material

**Fig. S3**. Negative frequency‐dependent selection for select pairs of resistant and sensitive isolates.

Supplementary Material

**Fig. S4**. Productivity of drug‐containing and drug‐free patches become similar by days 20 and 40.

**Fig. S5**. Plot of (final frequency of resistance – initial frequency of resistance) versus initial frequency of resistance.

**Fig. S6**. Negative frequency‐dependence using Chevin fitness (maximum growth rate of evolved isolate ‐ ancestor) versus log_10_MIC.

**Fig. S7**. Negative frequency‐dependence using assumed starting frequencies.
