# Supplementary material for: The emergence, maintenance, and demise of diversity in a spatially variable antibiotic regime
Source: Evol Lett. 2018 Mar 17;2(2):134–43. doi: 10.1002/evl3.43 (PMC6121846; doi:10.1002/evl3.43)
Supplement: Supplementary file 1 — Table S1. Mixed linear analysis of covariance for maximum growth rate in LB. Table S2. Mixed linear analysis of covariance for relative fitness (ω) of resistant types, with random effect of isolate pair nested in population. Table S3. Mixed linear analysis of covariance for maximum growth rate in [0.3 μg/mL] ciprofloxacin. Table S4. Mixed linear analysis of covariance for relative fitness (ω) of resistant types, with random effect of isolate pair nested in population. [file EVL3-2-134-s001.docx]

**Supplementary:**

**Table S1:** Mixed linear analysis of covariance for maximum growth rate in LB. Only populations with both resistant and sensitive isolated colonies were included for analysis. *X*^2^ and P values calculated by model comparison.

| **Effect** | **Term** | ***X^2^*** | **P** | **SD** |
| --- | --- | --- | --- | --- |
| Fixed | log_10_MIC | 22.1312 | 2.546e-06 |  |
|  | day | 6.1436 | 0.01319 |  |
|  | log_10_MIC*day | 4.1708 | 0.04113 |  |
| Random | Population (intercept) |  |  | 0.1394 |
|  | Population (slope) |  |  | 0.0104 |

**Table S2:** Mixed linear analysis of covariance for relative fitness (ω) of resistant types, with random effect of isolate pair nested in population. Only populations with both resistant and sensitive isolated colonies were included for analysis. *X*^2^ and P values calculated by model comparison.

| **Effect** | **Term** | ***X^2^*** | **P** | **SD** |
| --- | --- | --- | --- | --- |
| Fixed | initial frequency | 45.8185 | 1.299e-11 |  |
|  | day | 5.9514 | 0.01471 |  |
|  | initial frequency*day | 2.1507 | 0.14251 |  |
| Random | pair : population (intercept) |  |  | 0.06733 |
|  | pair : population (slope) |  |  | 0.04819 |
|  | Population (intercept) |  |  | 0.09806 |
|  | Population (slope) |  |  | 0.04550 |

**Table S3:** Mixed linear analysis of covariance for maximum growth rate in [0.3µg/mL] ciprofloxacin. Only populations with both resistant and sensitive isolated colonies were included for analysis. *X*^2^ and P values calculated by model comparison.

| **Effect** | **Term** | ***X^2^*** | **P** | **SD** |
| --- | --- | --- | --- | --- |
| Fixed | log_10_MIC | 16.8488 | 4.048e-05 |  |
|  | day | 0.7984 | 0.3716 |  |
|  | log_10_MIC*day | 0.2112 | 0.6459 |  |
| Random | Population (intercept) |  |  | 0.1262 |
|  | Population (slope) |  |  | 0.1842 |

**Table S4:** Mixed linear analysis of covariance for relative fitness (ω) of resistant types, with random effect of isolate pair nested in population. Estimated frequencies (from 40 random colonies) were substituted with assumed starting frequencies (i.e., 0.1, 0.5, and 0.9). Only populations with both resistant and sensitive isolated colonies were included for analysis. *X*^2^ and P values calculated by model comparison.

| **Effect** | **Term** | ***X^2^*** | **P** | **SD** |
| --- | --- | --- | --- | --- |
| Fixed | assumed starting frequency | 36.5932 | 1.455e-09 | Fixed |
|  | day | 4.4974 | 0.03395 |  |
|  | assumed starting frequency*day | 1.9320 | 0.16454 |  |
| Random | pair : population (intercept) |  |  | 0.06252 |
|  | pair : population (slope) |  |  | 0.03892 |
|  | Population (intercept) |  |  | 0.11190 |
|  | Population (slope) |  |  | 0.07648 |

**Fig. S1. SPAT selection regime.** Each population consisted of bacterial cultures propagated in two separate wells of 1.5mL LB broth and 1.5 mL ciprofloxacin(0.3µg/mL), where 750µL of each well was combined after 24 hours of growth and 20µL of the combined mixture (i.e., population) was then immediately transferred again to separate wells for the next growth cycle.

**Fig. S2.** **Coexistence of susceptible and resistant types maintained in SPAT treatment.** Evolved levels of resistance at day 40, measured as the log minimal inhibitory concentration (MIC) of ciprofloxacin, for isolated colonies for each SPAT population (**A)** and TEMP population **(B).** Blue axis indicates ancestral PA14 isolate resistance (MIC = 0.5µg/mL). Resistant isolates were defined as exhibiting an MIC exceeding 2µg/mL (log_10_(MIC) = 0.3), as indicated by the orange dashed line. Each data point represents a single isolated colony.

**Fig. S3. Negative frequency-dependent selection for select pairs of resistant and sensitive isolates.** Fitness of a resistant colony relative to its paired sensitive colony (ω) is plotted as a function of its frequency for isolates from SPAT population S4 (day 20) **(A),** and population S2 (day 40) **(B).** Solid colour lines depict linear regressions of individual resistant-sensitive isolate pairs, while pooled data regressions are shown by the dashed line. The frequency of resistance at equilibrium is given by the intersection of the regression line with relative fitness (ω) = 1.

**Fig. S4. Productivity of drug-containing and drug-free patches become similar by days 20 and 40.** Productivity of evolved populations, measured as the optical density at 600 nm after 24 hours of growth in the absence (LB - white boxes) or presence of [0.3µg/mL] ciprofloxacin (CIP - grey boxes) at day 0, 20 and 40. Bolded lines indicate median values, with surrounding boxes depicting first and third quartiles.

**Fig. S5. Plot of (final frequency of resistance – initial frequency of resistance) versus initial frequency of resistance.** This corresponds to the observed negative frequency-dependent selection depicted in Fig. 4.

**Fig. S6. Negative frequency-dependence using Chevin fitness (maximum growth rate of evolved isolate - ancestor) versus log_10_MIC.** The results using the Chevin measure of fitness are consistent with our results using the ratio measure of fitness, as depicted in Fig. 4 (i.e., growth rate of evolved isolate / ancestor).

**Fig. S7: Negative frequency-dependence using assumed starting frequencies.** Estimated frequencies (from 40 random colonies) were substituted with assumed starting frequencies (i.e., 0.1, 0.5, and 0.9).
