# Supplementary figures and images for: The emergence, maintenance, and demise of diversity in a spatially variable antibiotic regime
Source: Evol Lett. 2018 Mar 17;2(2):134–43. doi: 10.1002/evl3.43 (PMC6121846; doi:10.1002/evl3.43)

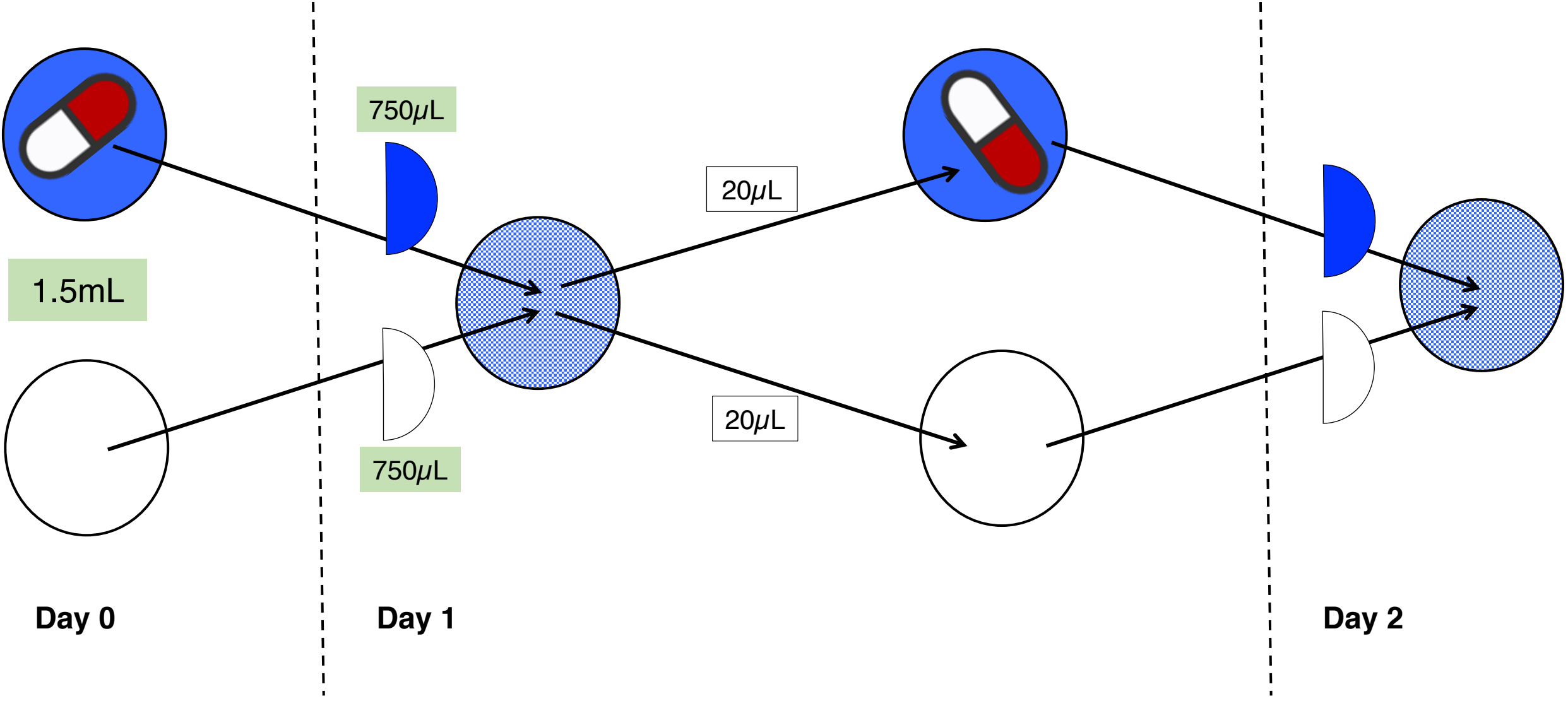

Supplement: Supplementary file 2 — Fig. S1. SPAT selection regime. [file EVL3-2-134-s010.pdf]

**A**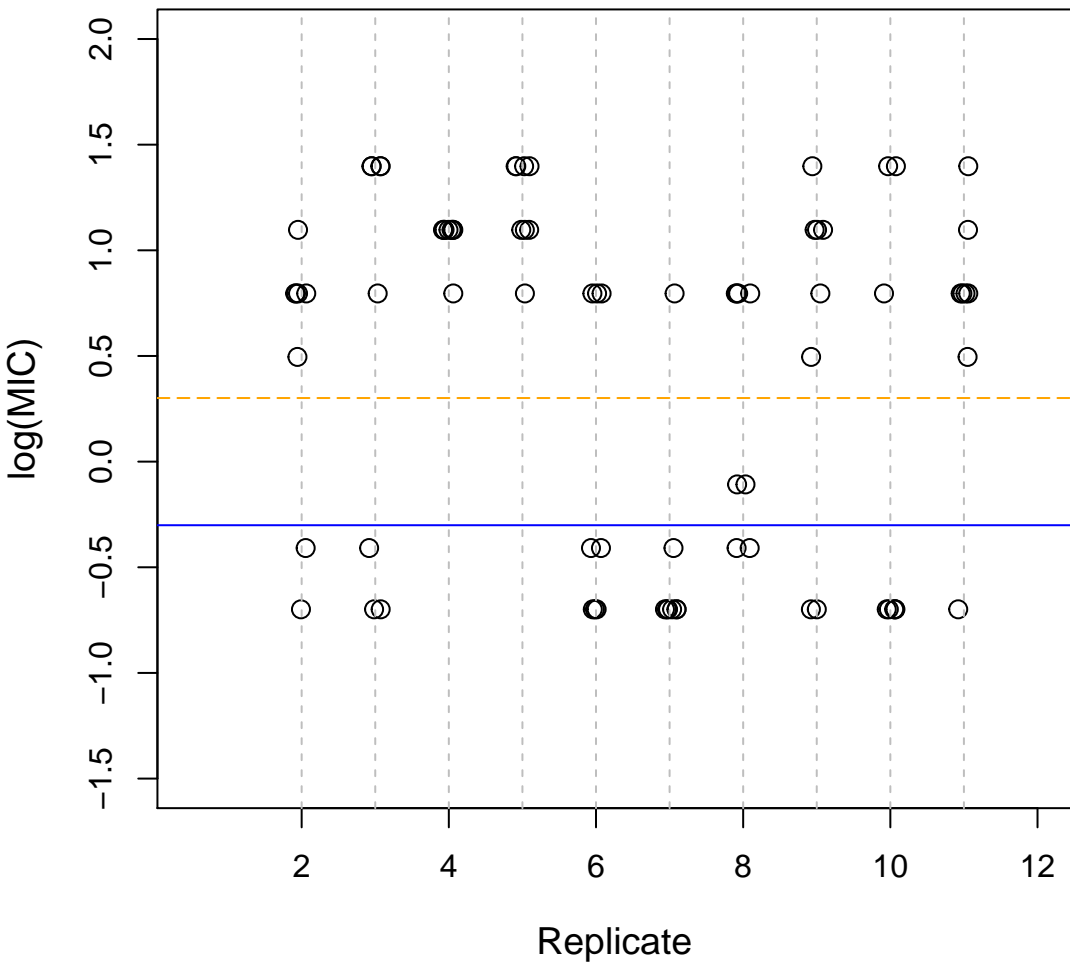

Supplement: Supplementary file 3 — Fig. S2. Coexistence of susceptible and resistant types maintained in SPAT treatment. [file EVL3-2-134-s002.pdf]

**B**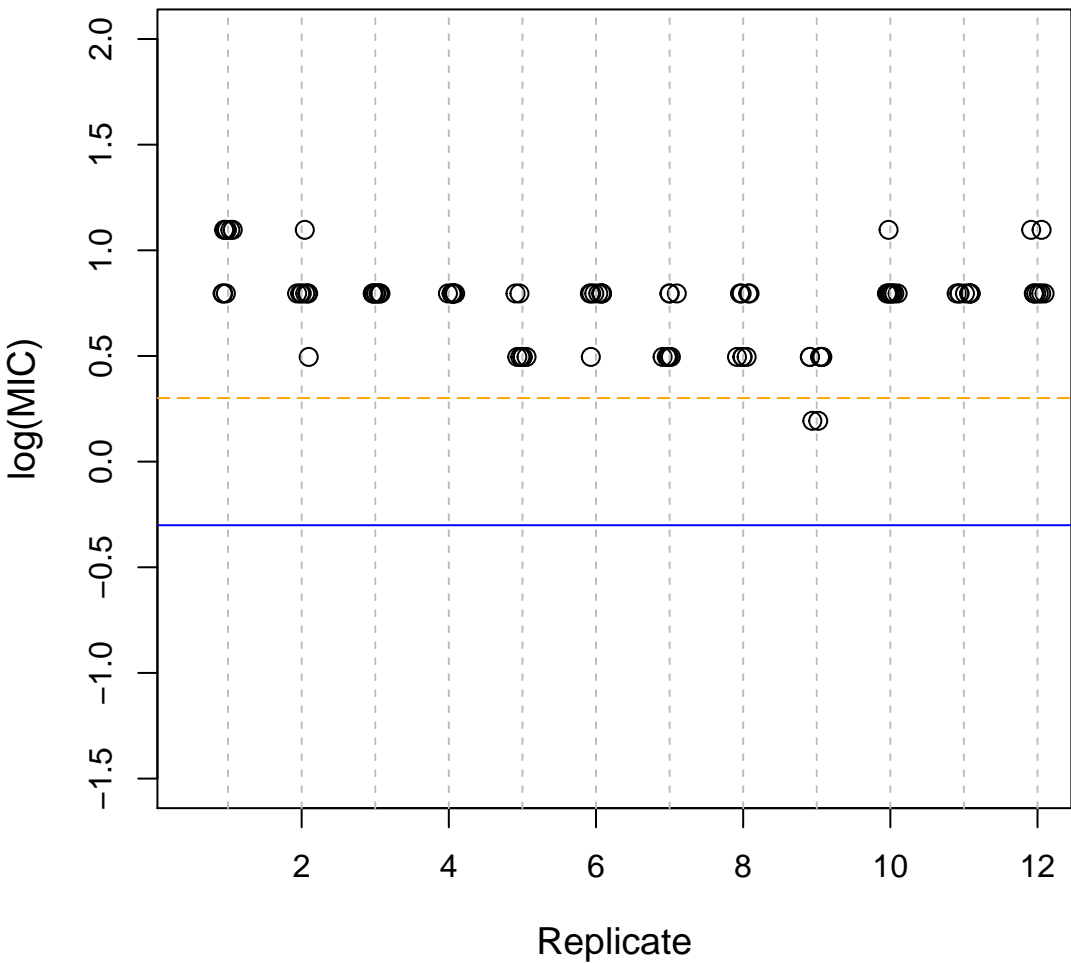

Supplement: Supplementary file 4 — Supplementary Material [file EVL3-2-134-s003.pdf]

**A****Relative fitness of resistance****S.4 day20**

h-b  
c-f  
h-a  
e-d

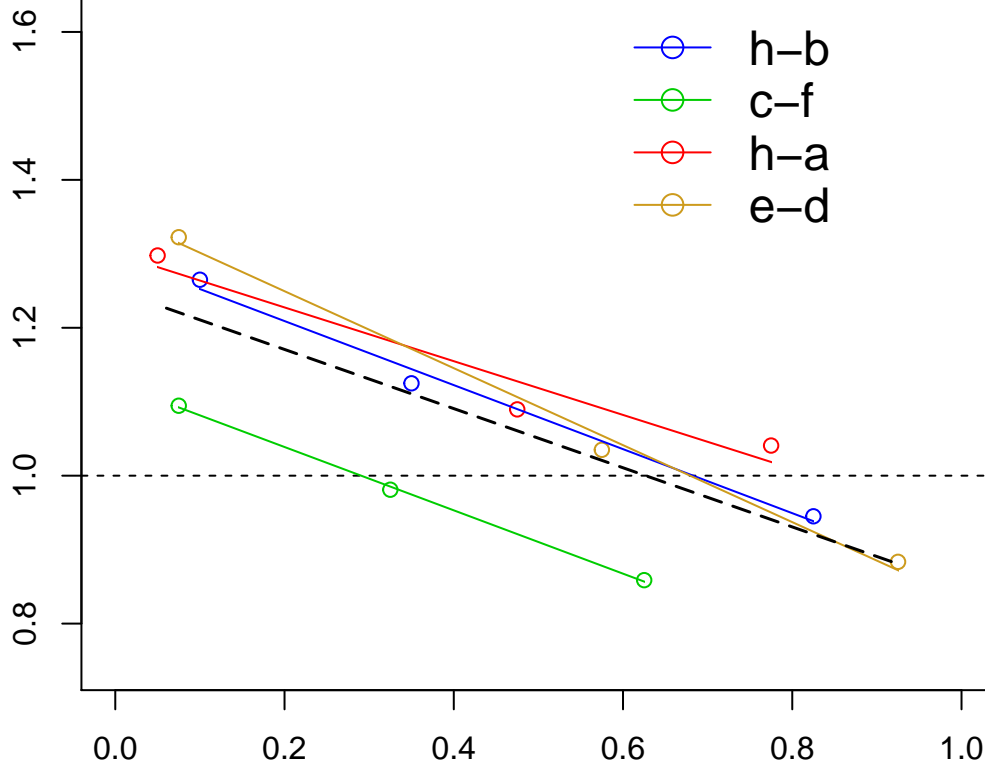**Frequency of resistance**

Supplement: Supplementary file 5 — Fig. S3. Negative frequency‐dependent selection for select pairs of resistant and sensitive isolates. [file EVL3-2-134-s004.pdf]

**B****Relative fitness of resistance****S.2 day40**

a-h  
c-g  
b-h  
f-h

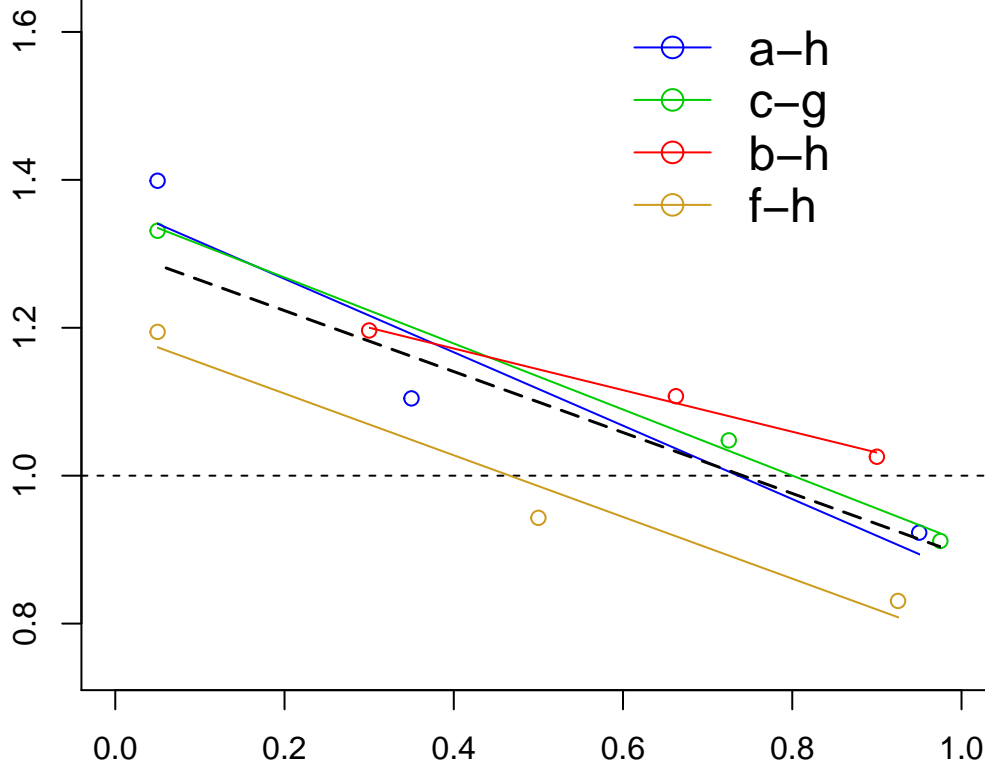**Frequency of resistance**

Supplement: Supplementary file 6 — Supplementary Material [file EVL3-2-134-s005.pdf]

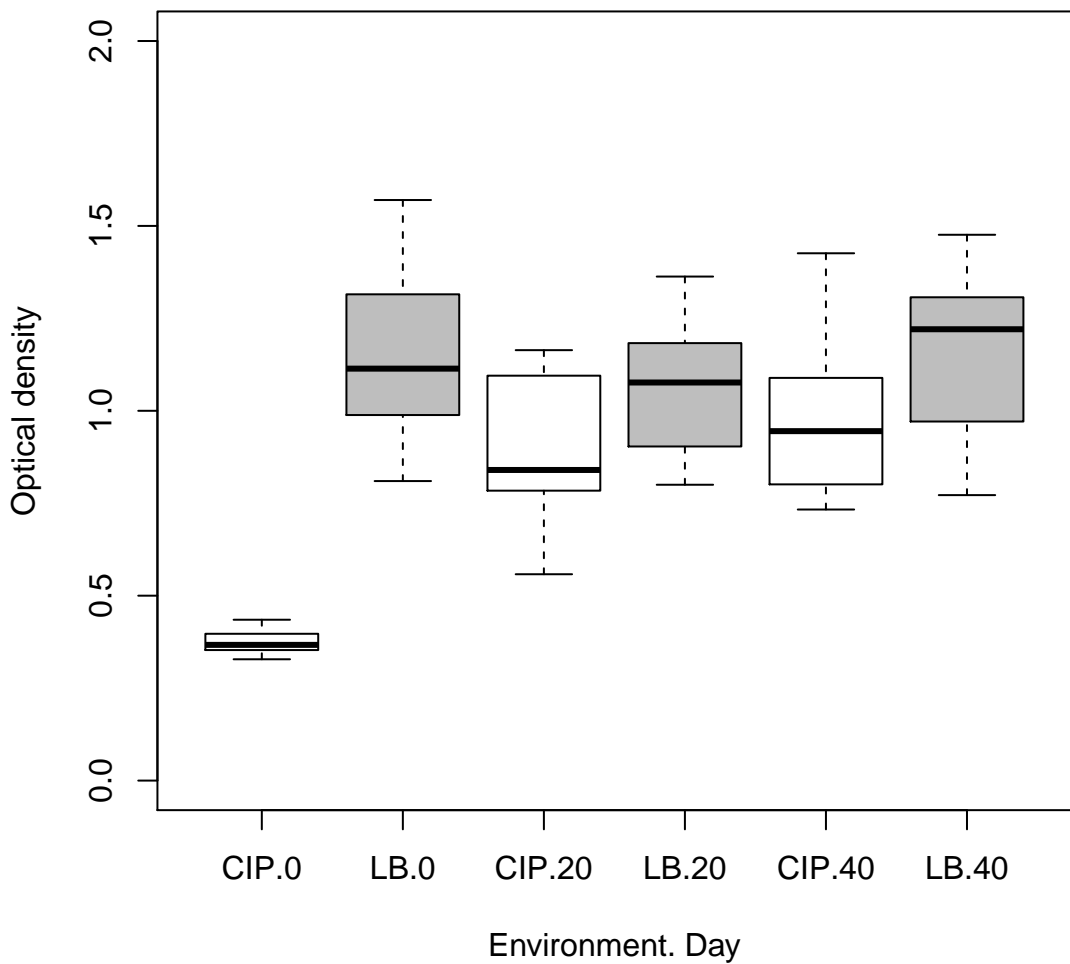

Supplement: Supplementary file 7 — Fig. S4. Productivity of drug‐containing and drug‐free patches become similar by days 20 and 40. [file EVL3-2-134-s006.pdf]

**Change in resistance frequency**

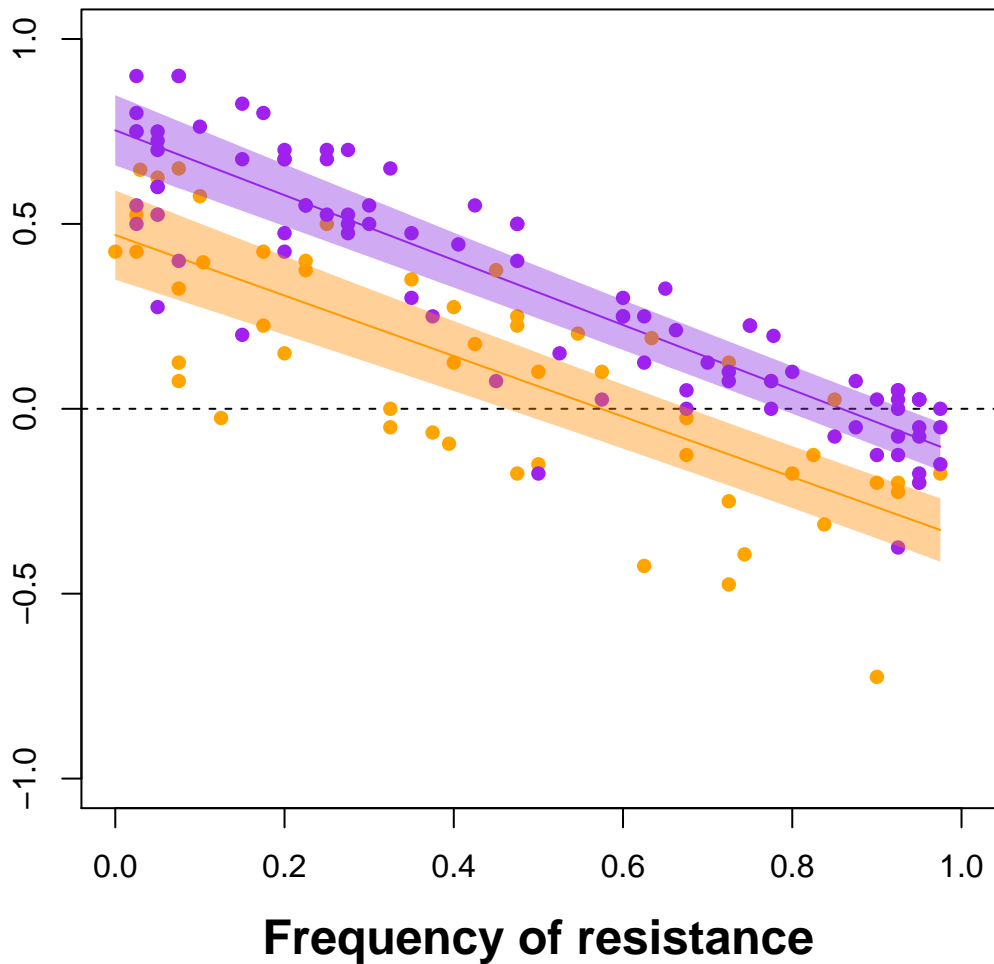

Supplement: Supplementary file 8 — Fig. S5. Plot of (final frequency of resistance – initial frequency of resistance) versus initial frequency of resistance. [file EVL3-2-134-s007.pdf]

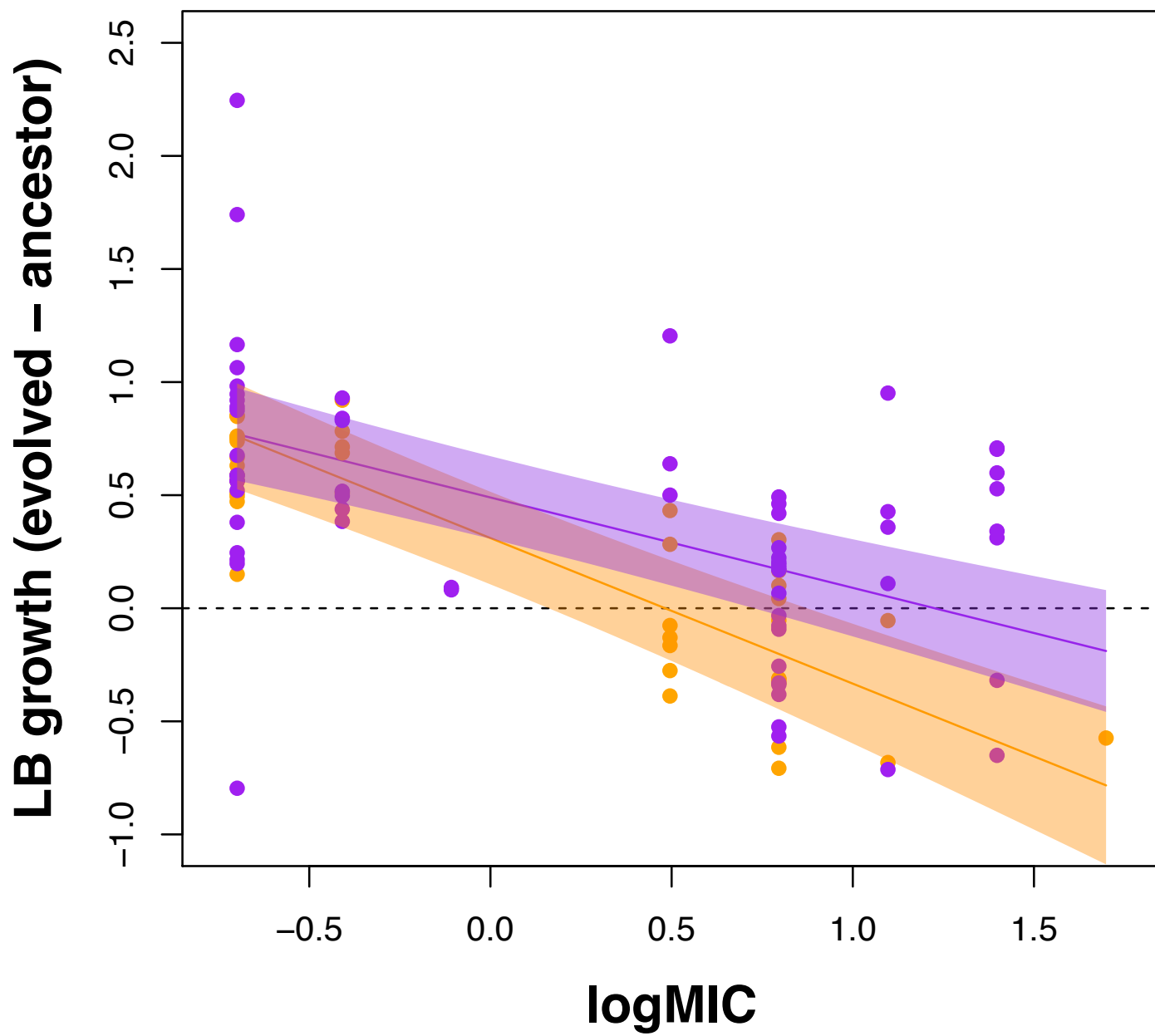

Supplement: Supplementary file 9 — Fig. S6. Negative frequency‐dependence using Chevin fitness (maximum growth rate of evolved isolate ‐ ancestor) versus log10MIC. [file EVL3-2-134-s008.pdf]

**Relative fitness of resistant type**

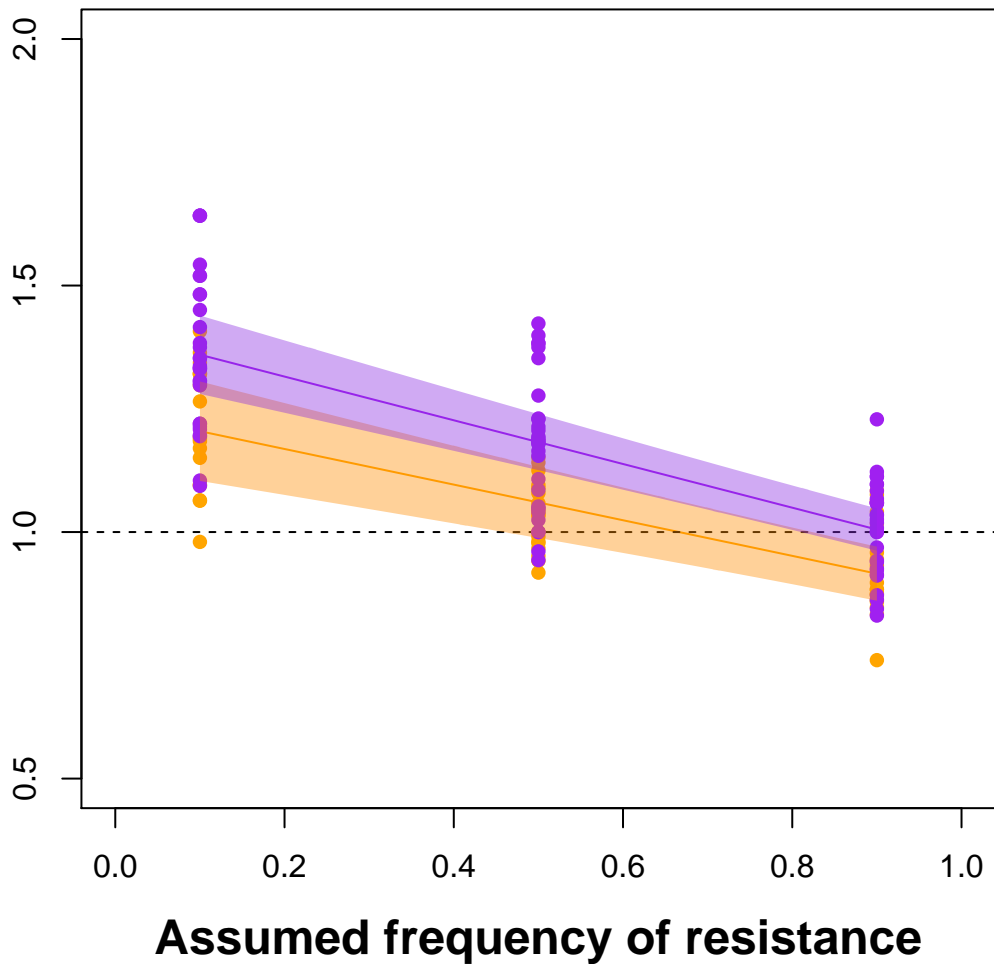

Supplement: Supplementary file 10 — Fig. S7. Negative frequency‐dependence using assumed starting frequencies. [file EVL3-2-134-s009.pdf]
